# P-1328. Contribution of Antimicrobial Resistance Data of NARMS Retail Meat in the Investigation of Persistent Strain of *Salmonella* Hadar Infections, Tennessee, 2021-2023

**DOI:** 10.1093/ofid/ofae631.1506

**Published:** 2025-01-29

**Authors:** Samir Hanna, Katie Garman, John R Dunn

**Affiliations:** Tennessee Department of Health, Nashville, Tennessee; Tennessee Department of Health, Nashville, Tennessee; Tennessee Department of Health, Nashville, Tennessee

## Abstract

**Background:**

Reoccurring, Emerging, and Persisting (REP) strains of *Salmonella* have caused illness for several years and are linked to multiple sources. Some REP isolates have been detected in food products like MDR *Salmonella* Infantis (REPJFX01) in retail chicken. Tennessee participates in the FDA’s National Antimicrobial Resistance Monitoring System (NARMS) Retail Meat Surveillance to monitor the antimicrobial resistance of retail meat enteric bacteria isolates. We reviewed the contribution of TN NARMS data in the investigations of multistate (MS) outbreaks caused by persistent strain of *S*. Hadar (REPTDK01).

**Methods:**

Tennessee submits every 20^th^*Salmonella* isolates from humans statewide, and all *Salmonella* isolates from retail meat (retail chicken, ground turkey, ground beef and pork products) purchased from 13 counties to NARMS. Sequenced isolates are uploaded to PulseNet, and resistance determinants are monitored. TN responds to CDC/FDA requests on isolates with persisting strains matching MS outbreaks.

**Results:**

REPTDK01 strain was detected in 4 ground turkey (GT) isolates from TN as part of 2 multistate outbreaks in 2021 (2102MLTDK-1) and 2023 (2302MLTDK-1). Over 95% of human and non-human isolates in both outbreaks were resistant to tetracycline, streptomycin, or both, and 0.2-0.4 % to ciprofloxacin and ceftriaxone respectively. Three of the 4 GT samples were purchased from grocery stores in different counties in 2021, and two of them had the same producer establishment number. USDA issued a public health consumer alert for over 211,406 pounds of raw GT products produced by the establishment. An additional GT sample was purchased in 2023 with a different establishment number. Consumption of GT was reported by 20 (60%) of 33 cases in 2021, while no signal of GT consumption among 55 cases in 2023. TN had no human cases in both outbreaks. However, a total of 49 cases from TN were linked to another three MS outbreaks of REPTDK01 associated with backyard poultry contact between 2019 and 2023 with one death in 2022.

**Conclusion:**

NARMS retail meat routine surveillance data contributes to a better understanding of source attributions of REP bacterial strains. Sharing of TN data with federal partners helped in traceback efforts, regulatory actions, and alerting the public.

**Disclosures:**

**All Authors**: No reported disclosures

